# Technical Considerations of Ozonated Oils in Medical Applications: A Narrative Review

**DOI:** 10.7759/cureus.83185

**Published:** 2025-04-29

**Authors:** Mandala Sathwik, Satyalakshmi Komarraju, Sathyanath D, Shrikanth Muralidharan

**Affiliations:** 1 Department of Ozone Therapy, National Institute of Naturopathy, Ministry of Ayush, Government of India, Pune, IND; 2 Department of Naturopathy and Yoga, National Institute of Naturopathy, Ministry of Ayush, Government of India, Pune, IND; 3 Department of Clinical Naturopathy, National Institute of Naturopathy, Ministry of Ayush, Government of India, Pune, IND; 4 Department of Research, National Institute of Naturopathy, Ministry of Ayush, Government of India, Pune, IND

**Keywords:** clinical use, conventional treatments, criegee reaction, ozonated oils, quality control

## Abstract

Ozonated oils, derived from the reaction of ozone with unsaturated fatty acids in vegetable oils such as coconut, olive, sunflower, and sesame, have gained significant attention in medical applications due to their recognized germicidal, immune-stimulating, and tissue-repairing properties. These stabilized ozonated oils release ozone gradually in the form of ozonides, which are stable and capable of providing prolonged therapeutic effects, mainly when applied topically. The germicidal properties of ozonated oils, primarily exerted through the oxidation of microbial cell walls, make them effective in treating various conditions, including skin infections, dermatitis, and fungal infections. Despite ozone's inherent instability, ozonated oils have demonstrated substantial therapeutic efficacy, including bactericidal, fungicidal, and virucidal effects. The clinical use of ozonated oils is expanding, offering a lower-cost, effective alternative to conventional treatments, especially in managing infections resistant to antibiotics. While their primary application remains topical, there is growing evidence of their positive effects when used orally, particularly in enhancing immune function and promoting tissue regeneration. This review examines the technical considerations for the medical application of ozonated oils, focusing on their stability, chemical characteristics, and therapeutic potential.

## Introduction and background

The clinical use of ozonated oils dates back to 1859, with early evidence supporting their therapeutic potential [1]. Ozonated oils are created by reacting ozone (oxygen) with unsaturated fatty acids in vegetable oils, producing oxidation products with germicidal (germ-killing), immune-stimulating, and tissue-healing properties. Some of the most commonly used ozonated oils are made from sunflower and olive oils, which have been applied to various medical conditions. These oils are used primarily on the skin, but recent research also shows that they may have beneficial effects when taken orally, helping to boost the immune system and aid in healing [2,3].

Dr. Velio Bocci, a leading researcher in ozone therapy, believes that ozonized oil, an affordable and simple treatment, could revolutionize how we treat skin ulcers and wounds, offering a more effective alternative to expensive pharmaceutical creams. He even predicted that ozone would become the “wonder drug” of the 21st century [4]. Although ozone is unstable, it can be stabilized in ozonated oils such as triozonides (a compound formed when ozone bonds with fatty acids). This process occurs by bubbling ozone gas through oils, resulting in a stable product that retains its healing properties for years if stored correctly. Today, ozonized oils are used to treat a variety of conditions, such as traumatic wounds, infections that don’t need oxygen to grow, cold sores, trophic ulcers (a type of skin ulcer that is difficult to heal), burns, cellulitis (a skin infection), abscesses, anal fissures, bedsores, fungal diseases, gum disease, vaginal infections, and even skin problems caused by cancer treatment. The growing evidence supporting their effectiveness makes ozonized oils essential for managing chronic and hard-to-treat infections. Given their wide range of medical uses, it is necessary to understand the key factors contributing to their healing power [4].

## Review

Ozonation process

The ozonation of edible oil is performed by bubbling the gas mixture (O₂/O₃) for some hours. Ideally, any oil compatible with human usage can be used for ozonation and further applied for medical therapy [5-8]. As stated above, olive oil is commonly used. However, a wide range of oils are being used, from coconut to sesame oil. This may be affected by people's preferences (for example, cultural norms of India may prefer coconut), and also based on the seasonal availability of the oil extract. A trend nowadays is to have a cold press machine (which is portable) and have the oil extracted and later processed in a clinical practice. Its economic feasibility is yet to be discussed in the literature. However, such practices can ensure the controlled quality of pure oil, which has more biological therapeutic effects [9,10]. 

The ozonation of vegetable oils involves bubbling ozone, typically produced by high-concentration industrial ozone generators, through the oil under controlled reaction conditions (Table 1). This process results in various compounds, including ozonides (1,2,4-trioxolanes) and peroxides, such as hydroperoxides (H₂O₂, polymer peroxides, and other organic peroxides) [5]. The quality and therapeutic efficacy of the ozonated oils are influenced by several critical factors [3].

**Table 1:** Factors affecting the ozonation process of vegetable oils

Parameter	Indication
Type of ozone generator	In determining the ozone concentration and flow rate during the process.
Ozonation conditions	Such as ozone concentration, flow rate of the gas, and duration of exposure.
Vegetable oil type and quantity vs. ozonation time	The greater the degree of unsaturation in oil, the more time is taken for ozonation. For example, sesame oil will take more time than olive oil or coconut oil [6]. On the contrary, others state that the greater the unsaturation, the quicker the ozonation. This needs further standardised comparison for commercial applications [7].
Ozonation temperature	An inverse relation exists. The preference is for a low temperature for this procedure as cooler temperature increases solubility [8].
Agitation of the reaction mixture	The tall barrel mixer and diffuser will be at the lowest bottom site possible, ensuring a longer contact period, consistent reaction, and optimal production of ozonated derivatives [8].
Use of medical-grade oxygen as the feed gas	The nitrogen content in ambient air can reduce ozonation efficiency and lead to undesirable side effects [9].

Chemistry of the ozonized vegetable oil

The Criegee Reaction 

The Criegee reaction is a key process in the ozonation of vegetable oils, mainly when ozone interacts with unsaturated fatty acids. It involves the formation of ozonides, which are key compounds in ozonated oils. In the Criegee reaction, ozone reacts with the double bonds in unsaturated fatty acids, such as those found in vegetable oils. This reaction leads to the formation of a molozonide, a highly unstable intermediate, which then decomposes to produce ozonide-stable compounds (trioxolanes) that play a crucial role in the therapeutic properties of ozonated oils [3]. The formation of ozonides is responsible for the germicidal, immune-stimulating, and tissue-restoring effects observed in ozonated oils [4,5,10-12].

The ozone reaction with vegetable oils primarily targets the carbon-carbon double bonds in the unsaturated fatty acids. This reaction forms lipid peroxides, ozonides, and several oxygenated compounds such as aldehydes, peroxides, and polyperoxides [13]. These compounds may contribute to the broad antimicrobial activity observed in ozonized oils. The Criegee mechanism is essential because it governs the reaction kinetics and the overall stability of the ozonated product [14]. The degree of unsaturation of the fatty acids in the oil influences how readily they react with ozone. Understanding this reaction is crucial in controlling the production of ozonated oils, ensuring their stability and potency for medical use [3].

Quality control and safety considerations in ozonated oil

The efficacy and safety of ozonated oil are intrinsically linked to its quality control, with critical parameters that ensure its clinical effectiveness and safety. The indicators used to define the dosage and appropriate clinical application of ozonated oils are:

Peroxide Value (PV)

It is one of the indicators of the strength of the ozonation process [15]. The peroxide value (PV) represents the quantity expressed in milliequivalents of active oxygen contained in a 1000 g sample (mEq O2/kg) [3]. PV indicates the amount of peroxide in the ozonated oil and is expressed as the active oxygen amount per kilogram of sample (mmol-meq/kg) [7]. PV is a quantitative indicator of the concentration of peroxides within ozonated oil. PV also serves as a dosage criterion for ozonated oils. Ozonation increases PV [13]. These peroxides are pivotal to the oil's therapeutic properties, as they contribute significantly to its antimicrobial, healing, and cosmetic effects [16]. PV may be suitable for evaluating the stability of vegetable oil ozonides and is crucial for both commercial distribution and determining optimal storage conditions [17,18]. The measurement of peroxide concentration is critical in determining the therapeutic dose of ozonized oil. One of the challenges in the quality control of ozonated oil is the absence of a standardized method tailored for high peroxide levels. To minimize variability, it is recommended to use standardized methods, such as the International Scientific Committee of Ozone Therapy (ISCO_3_)-recommended method, to assay the PV [4]. PV plays a key role in determining the intended application of ozonated oils (Table 2).

**Table 2:** Peroxide value and its clinical applications in ozonated oil

Peroxide Value (mEq O_2_/kg)	Clinical Application	Reference
80 - 120	Cosmetics	[3]
200 - 400	Improves healing processes.	[3]
400	Oral administration (diseases of the gastrointestinal tract and following gastrointestinal surgery).	[16]
>400	Prominent germicidal effects.	[3]
400 – 600	Long-term applications (some hours) in the vaginal, rectal and nasal mucosa, trophic ulcers in the epithelialization phase, scalp and skin care. Wounds, trophic ulcers, and burns in the clear and frank granulation phase.	[16]
800	Very infected ulcers or wounds.	[16]
1200	Psoriasis, viral diseases, and fungal infestations of the skin.	[16]
>1500	Germicidal effect helps in all kinds of skin-related diseases, non-healing ulcers, burns, and dental pathology.	[19]
2400 - 2800	Short-term application (1-10 min) for the treatment of gum diseases, difficult-to-treat resistant viruses, and skin fungi.	[16]

Iodine Value (IV)

The iodine value (IV) is a key parameter for determining the degree of unsaturation in oils. It quantifies the amount of iodine (in grams) that reacts with the double bonds in 100 grams of oil. This measurement provides valuable insights into the chemical properties of the oil, particularly its reactivity and potential for undergoing specific chemical processes, such as ozonation. During ozonation, double bonds in the oil are consumed to form products like 1,2,4-trioxolane, and the IV is used to monitor this transformation. IV measures the total number of double bonds present in a sample. As such, it is a valuable chemical analysis for evaluating the reduction of double bonds during the oil ozonation process, providing insight into the formation of 1,2,4-trioxolane. Oil ozonation reduces the IV, indicating the consumption of double bonds by the oxy/ozone gas mixture [4,15].

The IV is inherently linked to the fatty acid composition of the oil. Oils rich in unsaturated fatty acids, such as oleic or linoleic acid, typically exhibit higher IVs, whereas oils with higher saturated fatty acid content will show lower values. This makes the IV a helpful indicator of the oil's chemical characteristics and potential applications. An important factor affecting the accuracy of IV measurement, particularly in ozonized oils, is viscosity. Ozonation increases oil viscosity, which can hinder the iodine reagent's access to the residual double bonds, leading to a potential underestimation of the IV. To ensure reliable results, viscosity-related limitations should be accounted for, either by modifying the assay conditions or by correcting for the effects of viscosity. This consideration is particularly relevant in producing and analyzing ozonized oils, where precision in measuring the IV is crucial for understanding the process dynamics and ensuring product quality [18].

Acid Value (AV)

The acid value (AV) is a critical quality parameter for ozonated oils, providing insight into the presence and concentration of free fatty acids. As triglycerides break down, these free fatty acids are produced during ozonation. Therefore, the AV increases after ozonation is observed [15].

The AV is quantitatively defined as the amount of potassium hydroxide (KOH) in milligrams required to neutralize the free fatty acids in 1 gram of oil. This parameter is a valuable indicator of the oil's oxidation level, directly affecting its therapeutic efficacy, stability, and safety profile. Monitoring AV is therefore indispensable in ensuring the consistency and reliability of ozonated oils for clinical or therapeutic applications. The ozonated oils with higher AVs had lower minimum inhibitory concentration (MIC) values (better antimicrobial activity) [4,15].

*Viscosity* 

Viscosity is a critical quality parameter in assessing ozonized oils, offering valuable information about the molecular transformations that occur during ozonation. Researchers can monitor how ozonation influences the oil's physical properties by measuring viscosity using precise instruments such as torsional oscillation or vibrating viscometers across temperatures. The viscosity decreases with temperature increase. The decrease in the degree of unsaturation and the increase in molar mass contribute to the increase in ozonated oils' viscosity [13].

Ozonated Oil Stability

The stability of ozonated oils is critical to ensuring their therapeutic efficacy and reliability in medical applications. Proper assessment of their stability is essential, particularly under typical storage conditions, to maintain their potency and expand their potential for commercial use.

Despite their importance, there is a notable gap in the literature regarding the long-term stability of ozonated vegetable oils in commercial formulations, such as Oleozon® (Centro de Investigaciones del Ozono (CENIC), Havana, Cuba). Stability evaluations frequently rely on measuring the PV to assess the integrity of ozonated oils. For instance, studies on ozonated sunflower oil, marketed as Neozone 4000 (Cosmoproject, Italy), have demonstrated stability for at least six months when stored under refrigeration or at room temperature. However, extended storage durations or exposure to elevated temperatures (e.g., 40°C) significantly reduce ozonide content, directly affecting the oil's therapeutic potential [20]. Degradation studies of ozonated oils, particularly those analyzed through advanced techniques like gas chromatography-mass spectrometry (GC/MS), have identified key degradation products, including furyl derivatives, aldehydes, and carboxylic acids. Aldehydes, in particular, are notable for imparting unpleasant odors and contributing to the rancidity of the oil.

Additionally, external factors such as light exposure and moisture can accelerate the breakdown of glyceryl-based ozonides. These findings underscore the necessity of storing ozonated oils under controlled conditions to minimize degradation and preserve their functional integrity [21]. Pioneering research by Moureu et al. examined the stability of ozonated high-oleic sunflower oil under a range of storage conditions, with temperatures spanning from −20°C to +37 °C over one to 12 months. The study revealed that ozonated oils stored at ambient or elevated temperatures underwent rapid chemical degradation, whereas those refrigerated or frozen retained stability for over a year. Interestingly, despite chemical changes during storage, the oil's antimicrobial activity against *Streptococcus uberis* remained intact. This retained activity was attributed to the formation of secondary bioactive compounds, such as nonanoic and azelaic acids, compensating for the loss of ozonides [21,22]. Ozonated olive oil (OZO), renowned for its unique properties, has outperformed other ozonated oils in terms of antimicrobial effectiveness and stability. OZO demonstrates a remarkable shelf life, maintaining its therapeutic potential for up to a year at room temperature and extending to three to four years under refrigeration [22,23]. This resilience under varied storage conditions highlights its suitability for therapeutic applications. In addition, the oils ozonated for more extended hours (100-200 hours) have been stated to remain stable for one year at room temperature and for two years in the refrigerator [19].

Studies in the past decades on the use of ozonated oil 

Ozonated oils have been extensively studied for their antimicrobial, anti-inflammatory, and wound-healing properties in various medical and dental conditions. Among these, ozonated extra virgin olive oil has been widely investigated for its effectiveness (Table 3).

**Table 3:** Studies in the past decades on the use of ozonated oil OZO: ozonated olive oil; PV: peroxide value; PI: plaque index; Gi: gingival index; DNBS: 2, 4-dinitrobenzene sulfonic acid; SOD: superoxide dismutase; GSH-Px: glutathione peroxidase; ROS: reactive oxygen species; BXO: balanitis xerotica obliterans

Author	Type of oil used for ozonation	Ozonated oil with peroxide value used in mEq/O_2_ kg	Outcome
Crastechini et al. [24]	Extra virgin olive oil	900	Ozonized oil may be a new alternative for the control of denture stomatitis, showing similar clinical performance when compared to sodium bicarbonate.
Campanati et al. [25]	Type of oil not mentioned	-	Ozonated oil, topically applied for 12 weeks, seems to be as effective as hyaluronic acid in reducing symptoms related to skin burns, but it could be more effective in preventing post-lesional hyperpigmentation.
Ginel et al. [26]	Sunflower oil	950	Daily topical application of ozonated sunflower oil over the course of one week improved the healing of acute, full-thickness skin wounds in chelonians.
Bulani et al. [27]	Type of oil not mentioned	-	Ozone oil showed a significant reduction in the clinical parameters of PI and GI, similar to chlorhexidine gel.
Abu-Gharbieh et al. [28]	Pure virgin oil (oral administration)	400	OZO protects against 2,4-dinitrobenzene sulphuric acid DNBS-induced colonic mucosal damage in rats, likely by boosting antioxidant enzymes like SOD and GSH-Px, which help neutralize ROS.
Sechi et al. [29]	Sunflower oil	-	Mycobacteria are more susceptible to Oleozon (ozonated sunflower oil) than the other bacteria (*Staphylococci, Streptococci, Enterococci, Pseudomonas, *and* Escherichia coli*) tested.
Beltran et al. [30]	Olive oil (6-hour, 12-hour ozonated) and venadillo oil	(642.53, 703.7) and 892.12	Both ozonized venadillo and olive oils could serve as natural alternatives for treating bacterial infections.
Bianco et al. [31]	Ozosan® (Sanipan, Clivio, Italy)	-	Ozone oil suspensions demonstrated efficacy in non-invasive osteoradionecrosis treatment, likely due to their ability to stimulate local revascularization, antibacterial effects, and good protocol tolerability.
Menendez et al. [32]	Oleozon® (Centro de Investigaciones del Ozono (CENIC), Havana, Cuba)	-	Topical Oleozon® showed superior effectiveness in treating onychomycosis compared to ketoconazole, with no observed side effects.
Materni et al. [33]	Sunflower oil-based ozone gel Ozosan^®^ (Sanipan srl, Clivio (VA), Italy)	-	Ozosan^®^ reduces the incidence of dry socket (DS) following inferior third molar extraction.
Xiao et al. [34]	Oil (type not mentioned) with camellia oil	-	Ozone oil treatment significantly accelerated wound healing compared to the control group, with wound areas decreasing faster and recovery efficiency showing a clear time-dependent pattern.
Gültekin et al. [35]	Olive oil	-	OZO effectively attenuates both macroscopic and pathological symptoms of acute radiation proctitis in rats.
Lu et al. [36]	Type of oil not mentioned	-	Ozonated oil effectively suppresses inflammation in an atopic dermatitis murine model by reducing Th2-dominant cytokine responses and increasing IL-10 expression.
Currò et al. [37]	Ozoile^® (^Erbagil S.r.l, Benevento, Italy) Ozonated olive oil with vitamin E acetate		Treatment with Ozoile® effectively reduced inflammation and stimulated tissue regeneration mechanisms in the foreskin of children affected by BXO.
Varol et al. [38]	Olive oil 1 (OZO1), olive oil 2 (OZO2)	1352, 1053	OZO1 and OZO2 demonstrated effectiveness against four strains of *S. capitata*, with OZO1 showing superior activity. The study also confirmed that certain yeast strains resistant to other antifungal agents were susceptible to OZO. Additionally, the PV of OZO plays a crucial role in its antifungal activity. It is essential to analyze the PV of OZO preparations, as those with low PVs should not be used for treating yeasts.
Yousefi et al. [39]	Sunflower and sesame oil	-	Sesame and sunflower oils, especially when ozonated, can serve as effective inhibitors of *S. aureus* growth and may be useful in pharmaceutical formulations for wound and burn healing.
Tan et al. [40]	Type of oil not mentioned	-	Ozonated oil treatment for stable psoriasis demonstrated safety and effectiveness, with efficacy comparable to glucocorticoid topical preparations.
Aziza et al. [41]	OZO cream	-	Both OVO ointment and povidone-iodine 10% wet dressing effectively promoted healing of grade I diabetic foot ulcers, with OZO ointment showing superior healing effects.
Tara et al. [42]	OZO		Both ozone and clotrimazole significantly reduced symptoms and led to negative cultures (P < .05). No significant differences were observed between groups for itching, leucorrhea, or culture results (P > .05). However, clotrimazole significantly reduced burning sensation more than ozone (P < .05).
Montevecchi et al. [43]	Ozonated extra virgin olive oil - (Novox®, MOSS S.r.l., Lesa - Novara, Italy)	560/590	O3-Oil showed significantly greater growth inhibition diameters (P < 0.01) than chlorhexidine digluconate (CHX) and povidone-iodine (PVP-I) at all dilutions for both strains. CHX lost efficacy beyond a 1:32 dilution. At the highest dilution, inhibition diameters were: *Staphylococcus aureus*: 20.67±0.58 mm (O3-Oil) vs. 15.33±0.58 mm (PVP-I), *Porphyromonas gingivalis*: 19.00 mm (O3-Oil) vs. 13.67±0.58 mm (PVP-I).
Patel et al. [44]	Type of oil not mentioned	-	The study assessed the therapeutic effects of topical ozonated oil on the early healing of free gingival graft surgical sites. Cytological results showed significant improvement in epithelial healing at 7, 14, and 21 days, and two, three, and eight months postoperatively in the ozone group compared to the control group (P < 0.001).
Vadhana et al. [45]	Ozonated sesame oil	-	Oil pulling therapy using sesame oil and ozonated sesame oil showed a significant improvement in oral hygiene.
Patel et al. [46]	OZO	-	Topical OZO monotherapy showed significant improvement in clinical and microbiological parameters over time (P < 0.001), with no documented side effects.
Anzolin et al. [47]	Type of oil not mentioned		Topical application of ozonated oil resulted in pain relief in all groups except the placebo group (severe osteoarthritis, degree 4). P-value for treatment vs. placebo: 0.021 and 0.345, respectively.
Haghighizadeh et al. [48]	Lotion of OZO	2439	All patients with pediculosis of the head treated with OZO lotion were cured after one week, compared to those treated with permethrin, who required more treatment.
Solovastru et al. [49]	Spray formulation of ozonated oil (type not mentioned) and α-bisabolol combination	-	In a study of 29 patients with chronic venous leg ulcers, those treated with ozonated oil and α-bisabolol showed a significantly higher rate of complete ulcer healing (25% vs. 0%). The changes in ulcer surface area were significant only for the ozonated oil and α-bisabolol formulation (P < .05).
Augello et al. [50]	Extra virgin olive oil	2700-2900	OZO demonstrated potent antifungal activity against *C. albicans* by reducing cell viability, inhibiting hyphal and biofilm formation, and triggering oxidative stress and autophagy pathways.
Paoli et al. [51]	Type of oil not mentioned	-	The use of ozonized oil during sports massage significantly increased blood lactate removal, improved performance, and reduced the perception of fatigue in cyclists following 3 Wingate tests.
Ramírez-Hobak et al. [52]	OZO	-	OZO applied every 12 hours for 10 days resulted in the disappearance of coral-red fluorescence, erythema, fissures, pruritus, and maceration in all patients with interdigital foot erythrasma. Cure was achieved in 100% of patients, with only two cases of persistent scaling, showing similar efficacy to oral erythromycin.

Crastechini et al. [24] demonstrated that ozonized olive oil could be a viable alternative to sodium bicarbonate in managing denture stomatitis, as both treatments exhibited similar clinical outcomes. Abu-Gharbieh et al. [28] studied the oral administration of ozonated pure virgin olive oil. They found that it protected against colonic mucosal damage by enhancing antioxidant enzyme activity, which helps neutralize harmful reactive oxygen species. Gültekin et al. [35] explored its application in radiation-induced bowel injury, significantly reducing macroscopic and pathological symptoms. In a comparative study, Tara et al. [42] found that OZO was as effective as clotrimazole in treating vaginal infections, with both treatments leading to negative cultures. However, clotrimazole provided more significant relief from burning sensations. Montevecchi et al. [43] examined the antimicrobial activity of ozonated extra virgin olive oil. They reported it exhibited superior growth inhibition against *Staphylococcus aureus* and *Porphyromonas gingivalis* compared to chlorhexidine and povidone-iodine. Haghighizadeh et al. [48] investigated its use in treating head lice and found that all patients were cured within one week, outperforming permethrin. The antifungal potential of OZO was highlighted in a study by Augello et al. [50]. It demonstrated its ability to reduce *Candida albicans* cell viability, inhibit hyphal formation, and trigger oxidative stress pathways. Ramírez-Hobak et al. [52] examined its application in treating interdigital foot erythrasma. They found a complete cure in all patients within ten days, with results comparable to oral erythromycin.

Ozonated sunflower oil has also been examined for its therapeutic benefits. Ginel et al. [26] found that its topical application accelerated the healing of acute, full-thickness skin wounds, making it beneficial for wound management. Sechi et al. [29] explored its antimicrobial properties and reported that *Mycobacteria* were more susceptible to ozonated sunflower oil than other tested bacterial strains. Materni et al. studied its dental applications and found that sunflower oil-based ozone gel significantly reduced the incidence of dry sockets following molar extractions [33]. Yousefi et al. examined combined effects of ozonated sunflower and sesame oils, finding that they exhibited vigorous antibacterial activity against *Staphylococcus aureus*, suggesting their potential for wound and burn healing [39].

Sesame oil has also been studied for its medicinal benefits when ozonated. Vadhana et al. [45] investigated its use in oil-pulling therapy and found that ozonated sesame oil improved oral hygiene. Yousefi et al. [39] highlighted its strong antibacterial effects, particularly against *Staphylococcus aureus*, which is commonly associated with wound infections.

Several studies have examined ozonated oils without specifying the base oil used. Campanati et al. [25] reported that topically applied ozonated oil was as effective as hyaluronic acid in reducing burn symptoms and preventing post-lesional hyperpigmentation. Bulani et al. [27] highlighted its ability to reduce plaque and gingival inflammation, showing results comparable to chlorhexidine gel. Lu et al. [36] demonstrated its effectiveness in alleviating inflammation in an atopic dermatitis murine model by modulating immune responses. Tan et al. [40] reported that ozonated oil was as beneficial as glucocorticoid topical treatments in managing psoriasis. Anzolin et al. [47] explored its role in osteoarthritis pain relief, showing significant pain reduction in all groups except those with severe disease. Solovastru et al. [49] examined a combination of ozonated oil and α-bisabolol, which significantly increased the rate of complete ulcer healing in patients with chronic venous leg ulcers. Paoli et al. [51] studied its effects on sports recovery, reporting that ozonated oil used during massage enhanced blood lactate removal and reduced fatigue perception in cyclists.

Ozonated oils, particularly those derived from olive and sunflower oil, have demonstrated significant therapeutic potential across various medical applications. Their ability to combat infections, promote wound healing, and reduce inflammation makes them promising alternatives or complementary treatments for conditions such as burns, ulcers, gingival diseases, and dermatological disorders. While current findings support their effectiveness, further clinical research is necessary to establish standardized treatment protocols, assess long-term safety, and explore their full therapeutic potential.

In summary, the stability of ozonated oils is influenced by multiple factors, including the type of oil, storage temperature, and exposure to environmental conditions. Refrigeration and careful handling significantly enhance the shelf life of these oils, ensuring their sustained therapeutic benefits. Continued research on stability dynamics and degradation pathways is vital to optimize their storage and expand their commercial and medical applications.

## Conclusions

Ozonated oils have emerged as a promising therapeutic option for managing infectious diseases, offering a cost-effective and versatile alternative to conventional treatments. Their broad-spectrum antimicrobial activity has demonstrated efficacy comparable to or surpassing traditional antibiotics, with a notably lower risk of adverse effects. Key bioactive compounds generated during the ozonation process, such as ozonides, exhibit multiple beneficial properties, including germicidal effects, immune system modulation, and enhanced tissue repair, making them suitable for diverse clinical applications. One of the key advantages of ozonated oils is their stability, which supports the development of standardized formulations for therapeutic use. However, ensuring consistent safety and efficacy requires rigorous quality control measures. Accurate characterization of their chemical and physical properties, such as PV and other indicators of ozonation levels, is essential for maintaining therapeutic reliability. Ozonated oils are utilized across medical fields, including dermatology, dentistry, ophthalmology, and gynecology. There is also growing interest in their application for oral health, supported by emerging clinical evidence. However, despite these advancements, large-scale, well-designed clinical trials are required to substantiate their broader utility and establish standardized formulations for widespread adoption. With their unique combination of antimicrobial, immunomodulatory, and tissue-repair properties, ozonated oils hold immense potential to address the growing challenge of antimicrobial resistance and enhance patient care across various specialties.
